# Self-Labelling, Causal Attributions and Perceived Stigma in People Negatively Affected by Gambling

**DOI:** 10.1007/s10899-025-10433-8

**Published:** 2025-10-23

**Authors:** Tyler McGinlay, Paul Delfabbro, Daniel King

**Affiliations:** 1 School of Psychology, University of Adelaide, Adelaide, SA Australia; 2 College of Education, Psychology and Social Work, Flinders University, Adelaide, SA Australia

**Keywords:** Stigma, Labelling, Attributions, Problem gambling

## Abstract

This study examined self-labelling, stigma and causal attributions in a sample of 300 people who had currently, or previously experienced, substantial gambling-related problems. Specific aims were to compare people’s use of more clinical labels with public health labels relating to gambling harm and to examine whether stigma was stronger in people who made more internal attributions and who adopted clinical labels. The results showed that people rarely adopted public health terminology relating to gambling harm either in self-description or when referring themselves to others. Clinical terms (addicted, problem, compulsive) were commonly endorsed as self-labels, but only ‘addicted’ was commonly used when referring to themselves to others. Stigma and clinical labelling were stronger when people had more severe gambling problems, but stigma did not independently predict clinical label use and was lower if people made more internal attributions (i.e., gambling caused by their own actions). The findings support the importance of individual preferences and the careful use of appropriate language in public contexts to reduce stigma, but question whether the current emphasis on harm-related labels and the preoccupation in some papers with some clinical labels for gambling disorder may be misplaced.

## Introduction

A topic of growing interest in the gambling literature is stigma which refers to negative evaluations about people who are negatively affected by gambling (Barney et al., 2006; Hing et al., 2016; Hing & Russell, 2017). Stigma is known to be a multi-faced concept which can be public, perceived or anticipated, or personal or internalised. Public stigma occurs when the community holds negative views about people with gambling problems (e.g., that they can’t be trusted or are irresponsible). The consequences can include social distancing (Hing & Russell, 2017; Lloyd et al., 2025), negative stereotypes or strong negative emotional responses (e.g., fear). People with gambling problems can, in turn, be negatively affected when they perceive these views and so stigma can become internalized leading to a loss of self-esteem, shame, withdrawal and secrecy (Corrigan & Watson, 2002b; Galanis et al., 2025; Hing et al., 2016). Apart from the potential harm to individuals, stigma is also known to be one of the principal barriers for help-seeking (Donaldson et al., 2015; Evans & Delfabbro, 2005; Hing et al., 2016; Rundle et al., 2025).

Recognition of these problems has led researchers and policymakers to find solutions to address the problem of stigma (Brown & Russell, 2020). A particular area of interest has been strategies to reduce what are seen as potentially stigmatising ways of describing or ‘labelling’ people with gambling problems and the disorder itself (Wöhr & Wuketich, 2021). One of these has been to change the type of language used to describe people. Another has been a shift away from perspectives that might be seen to impute blame or responsibility to individuals on the assumption that this fosters shame and resentment (Delfabbro & King, 2020). For example, in relation to language, there has been moves to adopt what is termed ‘person first’ language that avoids the implication that a person is defined by the disorder. Problem gamblers have become ‘people with gambling harms’ or ‘People with hazardous or risky gambling’. Public health-focused writing has encouraged the avoidance of the term ‘problem’ in preference for terms such as ‘people with gambling harm’ or ‘people harmed by gambling’. It is hoped that this shift in terminology will increase help-seeking and make people more willing to admit (to services or others) that they have difficulties with gambling (Victorian Responsible Gambling Foundation, 2024). The shift also reflects sensible and important academic developments in the field, including the greater focus on public health approaches that emphasise the significance of harm rather than behavioural pathology in gambling policy and psychometric measurement (e.g., Browne & Rockloff, 2020; Langham et al., 2016). Under this approach, greater emphasis has been directed towards separating the measurement of harmful impacts from behavioural dependence and using terminology approach for these two related, but distinct, areas of focus.

Another form of ‘de-stigmatisation’ has been to reframe conceptualisations of the condition away from disciplinary approaches or labels that are seen to ‘pathologize’ the behaviour or which imply individual causality or blame (Brown & Russell, 2020; Penfold et al., 2024). Criticism is directed as such terms as ‘problem or pathological gambler’ or ‘gambling addict’, not only because of the lack of person-first language, but also because they imply that the person is unwell and may continue to display unhealthy behaviour in the future. Such a person is seen as stigmatised because the attribution is internal and stable. The person is ‘addiction prone’ (Penfold et al., 2024) because the condition is seen to arise from inherent or immutable characteristics, such as genetics, differences in neurological makeup or disposition, or family upbringing (Quigley et al., 2020).

As Quigley et al. argue, labels and causal beliefs can lead to stereotyping and prejudice, but does the evidence support a connection between labelling and stigma? The answer appears to be unclear. For example, a study by Palmer et al. (2018) asked members of the general public to rate the level of stigma associated with a description of symptoms as compared with symptoms + the label “gambling disorder” (the current DSM-5-TR classification). No difference in public stigma was observed. Quigley et al. (2020) surveyed both student and general public samples and found that four labels (problem gambling, pathological gambling, gambling disorder, and gambling addiction) attracted very similar attitude and stigma scores. They concluded: “Overall, most of the analyses support the conclusion that the label used to describe gambling disorder does not affect attitudes and stigma towards the condition.” (p. 1223). However, their study did not compare these clinical terms with more current public health alternatives such as “People harmed by gambling/Affected by gambling harm”). As a result, the study does not examine the potential impact of public health labelling on stigma. These studies also tried to examine whether various causal explanations derived from a 6-dimension Reasons for Depression measure predicted stigma. The results showed mixed results with both uncontrollable and controllable factors endorsed as explanations for the development of gambling disorder and the various related definitions. The study did not, however, specifically compare common public health or sociological (industry/product accessibility) or external factors with attributions relating to the person’s behaviour (i.e., a clear distinction of who was responsible).

In another study, Penfold et al. (2024) analysed *YouTube* posts and found evidence that framing the condition as an illness (‘addiction’) was beneficial in some contexts. In effect, it absolved the person from blame or implications of moral weakness or flaws of character. This framing helped some partners accept the condition as something capable of being addressed through treatment. They also noted that some people would use semi-religious or medical metaphors (‘a bug’, ‘inner devil’) which implied an internal cause, but one that was not necessarily controllable. In other words, the internal vs. external distinction in causality is much more complex than a simple: product/environment (‘the machine/industry made me do it’) vs. person (‘I am responsible’) distinction. In fact, there may be individual differences in how people use and respond to different language.

Another relevant study was conducted by Lloyd et al. (2025) who asked a public sample of 3567 people in the UK to rate a series of vignettes describing a person with gambling problems and found greater social distance (e.g., not wanting to associate with) when the person had a gambling problem vs. no problem. They also examined a range of potential causal factors including: bad character’; ‘chemical imbalance in the brain’; ‘stressful life circumstances’; ‘genetic/inherited problem’; ‘god’s will’; and ‘the way they were raised’. Higher social distance ratings were given when the gambling was attributed to bad character (internal attribution) and an ongoing problem (stable attribution). Once again, the study did not compare broader public health or sociological explanations for gambling-problems.

## The Present Study

Much of the present public-health focus on gambling has involved a concerted shift away from clinical labels and explanations for gambling problems to a greater focus on gambling harms with implications for how gambling disorder is described and the publishability of papers using clinical languageHowever, relatively little research has examined how people who have experienced gambling problems describe themselves and how this relates to perceived stigma (both personal and public). Nor has there been an examination of how people respond to more public health-oriented terminology as compared with more clinical labels. Finally, no studies appear to have given respondents a clear attributional choice between external causes (e.g., industry vs. individual actions). Accordingly, this study had several aims: (a) To examine how people negatively affected by gambling describe their condition to themselves and to others; (b) To examine people’s attributions for the causes of their problems with gambling, in particular, the relative importance of their own actions as opposed to external factors; (c) To examine the relationship between personal and public stigma and the labels which people use to describe their problem to others; and (d) whether people who have more internal attributions for their problems report greater perceptions of stigma. Although some of these research questions were exploratory due to the lack of guiding research studies on public health labelling, several hypotheses were advanced at the commencement of the research. It was hypothesized that people who had more internal attributions would adopt more pathology-based labels to describe their condition and would report greater stigma as a result of blaming themselves for their condition.

## Method

### Participants

The initial sample comprised 323 responses, but was reduced to 300 (165 men, 131 women, 1 other, 3 missing) after excluding very incomplete responses (no key dependent measures such as stigma, labelling completed) and those who did not meet the inclusion criteria of experiencing substantial gambling problems in the last 5 years wither based on the last 12 months PGSI or life-time NODS (see measures below). Of the 300, 86% met the problem criterion for at least 1 of the 2 measures. The rest scored 4+ (substantial problems) on the NODS-lifetime and/or moderate risk on the PGSI with most scores 5+. Participants were sourced from a variety of continents: Asia (*n* = 5), Africa (*n* = 99), North America (*n* = 17), South America (*n* = 6), Europe/UK (*n* = 159) and Oceania *n* = 12), with 2 missing data-points. Most of the sample were younger (18–35, 72.7%) with lower representation in the middle (36–45, 23.7%) and older age group (55+, 3%).

## Measures

### Demographic Information

Participants were initially asked to provide basic demographic information prior to the commencement of the survey. Age, gender, and continent of residence were collected from all participants.

### Gambling Behaviours

Participants were asked to specify whether they were currently experiencing a gambling problem or had within the last 5 years, and to indicate their preferred gambling activities on binary scales (Yes/No).

### Self-Described Labels

Participants were then asked several questions to identify the specific terminology they use to describe themselves as a gambler. (e.g., addicted to gambling or problem gambler). Individuals indicated on a 5-point Likert-scale their level of agreement that specific labels described their experiences with gambling during its most challenging times (e.g., problem gambler, social gambler, pathological gambler). They were then asked to indicate which of these labels they might use to describe themselves to others with multiple responses allowed (binary: endorsed/not endorsed responses). The key terms included: ‘problem gambler’; ‘Addicted to gambling’; ‘Victim of gambling harm’; ‘Social gambler’; ‘Person harmed by gambling’; ‘Pathological gambler’; and, ‘Compulsive gambler’.

### Causal Attributions

Participants were asked to indicate on a 5-point Likert-scale to what extent they agreed that the various factors were responsible for the negative effects they experienced as a result of gambling. They could attribute it to their own actions (internal) or external factors (the action of others, the accessibility of gambling products, the government, gambling advertisements, the industry [bookmakers and casinos]).

### NODS Measure of Problematic Gambling Behaviours (Lifetime Measure)

The National Opinion Research Center DSM Screen for Gambling Problems (NODS; Gerstein et al., 1999) is a 17-item measure used to screen for problem gambling behaviors or risk-factors within both clinical and research settings, based on the 10 diagnostic criteria for pathological gambling within the DSM-IV. The scale employs dichotomous style responses (yes/no), with a branching logic, whereby certain follow-up items are contingent upon the endorsement of initial screening questions. The NODS consists of items such as “have you ever tried to stop, cut down, or control your gambling?”, and “has there ever been a period when, if you lost money gambling one day, you would often return another day to get even?”. Individuals are scored out of 10, with the life-time classifications of substantial problems for scores of 3–4 and problem gambling for scores of 5+. The Cronbach’s Alpha in this sample was good (α = 0.82).

### The Problem Gambling Severity Index (PGSI)

The Problem Gambling Severity Index (PGSI; Ferris & Wynne, 2001) is a 9-item 4-point Likert-scale instrument used to determine the severity of an individual’s gambling problem throughout the last 12 months. Items are scored with responses options “never”, “sometimes”, “most of the time”, and “always” scored from 0 to 3 respectively. Scores on the measure range from 0 to 27, with scores of 0 indicating no problem gambling risk, 1–4 considered low risk, 5–7 signifying moderate risk, and scores of 8 or more indicative of problematic gambling behaviors. The Cronbach’s alpha coefficient was excellent (α = 0.91) in this study.

### The Gambling Perceived Stigma Scale (GPSS)

The Gambling Perceived Stigma Scale (GPSS; Donalson et al., 2015) is a 13 item 4-point Likert-scale measure used to determine an individual’s perceptions of societal attitudes towards problem gambling individuals. As a multidimensional psychometric measure, the GPSS captures two main subscales of stigma: ‘contempt’ and ‘ostracism’ (Donalson et al., 2015). Items are scored 1–4, (strongly disagree (1), somewhat disagree (2), somewhat agree (3), and strongly agree (4) with higher summed scores indicating greater levels of perceived social stigma. An example item within the ‘contempt’ dimension is “most people believe that problem gamblers are too lazy”, whilst the ‘ostracism’ subscale consists of items such as “many people would avoid a person who had a gambling problem.” Scores range from 13 to 52 with higher scores indicating greater stigma. The GPSS displayed excellent internal consistency within the present study (α = 0.91).

### The Gambling Experienced Stigma Scale (GESS)

The Gambling Experienced Stigma Scale (GESS; Donalson et al., 2015) is a 13-item, 4-point Likert-scale unidimensional measure used to determine an individual’s recognition and internalization of stigmatizing societal stereotypes and beliefs surrounding their own problem gambling behaviors. Items are scored 1–4, (strongly disagree (1), somewhat disagree (2), somewhat agree (3), and strongly agree (4), with higher summed scores indicating greater levels of experienced stigma. The scale consists of items including, “I don’t think I can be trusted because I gamble”, and “others think I am not worth the investment of time and resources because I am a gambler.” Scores range from 13 to 52 with higher scores indicating greater stigma. The Cronbach’s Alpha was excellent (α = 0.90) in this study.

## Procedure

Prior to the commencement of recruitment and data collection, ethics approval was obtained through the School of Psychology’s Human Research Ethics Subcommittee (HREC; approval number 24/53B). Individuals wishing to participate in the study were invited via a link which directed them to the survey preamble, participant information and consent form, and information pertaining to the storage of their information following submission. Eligible participants were then directed to the survey hosted by *Qualtrics*, which took an estimated 15 min to complete in full. Participants were invited to complete an online survey only if they were currently, or had in the past 5 years, considered themselves to have a gambling problem. Individuals who indicated that they did not meet these criteria were thanked for their time and excluded from further participation. Initially, 13 individuals were recruited through advertisements posted on various problem gambling support groups on Facebook, as well as Reddit subreddit r/problemgambling with the permission of moderators. To take part, people had to self-report as having a problem with gambling now and/or in the last 5 years that led them to consider seeking help. Due to the difficulties in recruitment, an additional 427 participants were recruited through online crowdsourcing platform, Proflic, with 130 of these submissions screened-out due to ineligibility. Individuals who completed the survey through Prolific were subsequently reimbursed for their participation at the standard rate of A$17.67/hr, or $0.20 for those deemed ineligible.

## Analytical Approach

The first two aims of the study were addressed by examining descriptive data (the prevalence of different label endorsement in the sample and the nature of causal attributions). Correlation analysis was used to examine the relationship between stigma scores and problem gambling severity and causality ratings. The examine how labelling related to differences in stigma scores, people were divided into either one of the pathology or clinical label only group vs. using at least one other softer label (i.e., a harm-based label or ‘social gambler’). Further, net attribution scores were calculated by averaging responses to internal and external attribution items separately and subtracting the external score from the internal score, producing an overall attribution score for each participant where greater positive values indicate stronger internal attributions.

## Results

### Gambling and Problem Gambling

Sports betting was the activity on which respondents reported spending the most money in the past 5 years (*n* = 107, 35.7%), followed by online casino gambling (*n* = 76,= 25.3%), lotteries (*n* = 38, 12.7%), and slot-games (*n* = 33, 11.3%). The mean score on the Problem Gambling Severity Index (PGSI) (*M* = 12.0, *SD* = 6.76) was well above the ‘problematic’ threshold of 8 suggested by the authors of the scale (Ferris & Wynne, 2001); 66% were currently ‘problem gamblers’ (Scores 8+) and 24% at least moderate risk (scores 3–7). The mean NODS-life-time score was 5.55 (*SD* = 1.95) which represents a borderline score between substantial problems and life-time problem; 85% had scores 3 + which indicates substantial life-time problems and 53% a score of 5 + suggesting a life-time problem.

### Terms used by Respondents to Label Themselves

Table 1 provides a comprehensive review of the labels which individuals indicated are descriptive of their relationship with gambling, as well as the terms participants indicated they use to describe themselves to others which may, or may not, correspond with the language the use for themselves. The results show that people are most likely to understand their condition to themselves as an addiction (Addicted), a gambling problem (Problem gambler) or compulsive gambler. People were reportedly less likely to view themselves as a person harmed by gambling (the common public health definition) and endorsement of the differently worded labels was similar. However, the results change when people report how they describe themselves to others. ‘Addicted’ is clearly the most commonly used term with ‘problem’ or ‘compulsive gambler’ a distant second. Only around 1 in 5 refer to the public health or ‘harm definitions. The clinical term ‘pathological gambler’ is rarely used. Overall, people are much less likely to use labels when describing themselves to others than when self-labelling. There is little evidence of adoption of the public health harm terminology.

**Table 1:** Labels used by respondents to describe themselves

Label	Self (*N*)*	%	To others (*N*)	%	
Problem Gambler	213	71.0	103	34.3	
Addicted to Gambling	228	76.0	168	56.0	
Victim of Gambling Harm	118	39.3	55	18.3	
Social Gambler	137	45.7	90	30.0	
Person Harmed by Gambling	149	49.7	55	18.2	
Pathological Gambler	114	38.0	32	11.0	
Compulsive Gambler	179	59.7	100	33.7	

*Sum of strongly agree and agree

All of the self-ratings were positively and significantly (*p* <.05) associated with problem gambling scores (PGSI): 0.34 for ‘problem gambler’; 0.35 for ‘addicted‘;.31 for victim of gambling harm;.32 for ‘Person harmed by gambling’; 16 for social gambler;.49 for ‘Pathological gambler’ and.38 for ‘Compulsive gambler’. Mean PGSI scores for those who endorsed the different labels for others tended to be highest for those who endorsed pathological gambling (15.0, *SD* = 6.6), Victim of gambling harm/harmed by gambling (15.4, *SD* = 7.2, 15.5, *SD* = 6.9); in the middle for problem gambler (13.4, *SD* = 6.7). addicted (14.0, *SD* = 6.7) and compulsive gambler (14.0, *SD* = 6.0) and lower for social gambler (11.1, *SD* = 6.3). The differences between the public health and more clinical categories were therefore small.

### Self-Reported Stigma

Table 2 provides the relevant descriptive statistics pertaining to the stigma measures. Mean scores on the two measures of stigma, the Gambling Perceived Stigma Scale (GPSS) and the Gambling Experienced Stigma Scale (GESS) were consistent with a similar study conducted by Leslie & McGrath (2024) using a sample of problem gambling individuals, which reported GPSS scores of *M* = 40.49, and GESS scores of *M* = 32.19.

**Table 2:** Descriptive statistics for stigma measures

	Theoretical Minimum Score	Theoretical Maximum Score	Actual Minimum Score	Actual Maximum Score	M	SD
GPSS	13	52	14	52	41.1	7.2
GES	13	52	13	52	31.8	8.4

GPSS = The Gambling Perceived Stigma Scale; GESS = The Gambling Experiences Stigma Scale

### Problem Gambling and Stigma

Table 3 shows the correlation between stigma and problem gambling. As expected, a strong positive correlation was found between the two measures of problem gambling severity, the NODS and the PGSI. The NODS was also strongly positively correlated with the GESS, and moderately positively correlated with the GPSS. The PGSI was strongly positively correlated with the GESS and moderately positively correlated with the GPSS. Scores on the GPSS were moderately positively correlated with scores on the GESS. These correlations suggest that individuals with more severe gambling problems reported greater levels of stigma on both the GPSS and the GESS.

**Table 3:** Pearson’s correlation of problem gambling measures and stigma

	NODS	PGSI	GPSS
PGSI	0.53**	-	
GPSS	0.40**	0.39**	-
GESS	0.65**	0.68**	0.51**

NODS = National Opinion Research Center DSM Screen for

Gambling Problems; PGSI = Problem Gambling Severity Index;

GPSS = The Gambling Perceived Stigma Scale; GESS = The Gambling

Experiences Stigma Scale

**Correlation significant at the 0.01 level

### Attribution for the Cause of Problems and Association with Labels used for Self

Inspection of the descriptive data for attributions indicated that respondents generally attributed their problems to their own actions (92.0%); the next most common response was the accessibility of gambling (77.0%); advertising (60.3%) and then the industry (48.9%). The action of others associated with the gambler (26.3%) or the government (28.0%) were both less endorsed. To examine how overall internal vs. external attributions related to labels, an overall attribution score was calculated = the rating for my own actions (internal) – Average of the 5 external ratings (the action of others, accessibility, the government, advertising, the industry). Higher scores therefore indicated a stronger willingness to attribute the harm to one’s actions vs. other external influences. This rating was found to be unrelated to all the clinical pathology ratings, but negatively related to Victim of gambling harm, *r*(296) = − 0.24, *p* <.001, and Social gambler, *r*(296) =−0.31, *p* <.001. This was not consistent with the hypothesis that more internal attributions would be associated with the adoption of more clinical labels. Two of the less clinical labels were, however, negatively related to a more internal attribution.

### Overall Attribution and Relationship with Psychometric Measures

The overall attribution (more internal responsibility or blame for outcomes) was found to be negatively related to NODS scores, *r(*296) = − 0.22, *p* <.01, PGSI scores, *r*(296) = − 0.15, *p* <.01, unrelated to GPSS (0.09), but negatively related to GESS, *r* (296) = − 0.23, *p* <.01. In other words, contrary to predictions, taking responsibility for the outcomes (i.e., attributing outcomes to one’s own actions) was associated with reduced stigma.

#### Labels used for Others and Self-Reported Stigma

Another area of interest was to examine whether people who adopted more clinical labels when describing themselves to others would report higher stigma scores. While conducting these comparisons, it was also important to determine whether this might be confounded by the severity of self-reported gambling problems. As Table 4 indicates, self-reported stigma was higher for people who used more clinical labels when describing themselves to others, but such people also tended to report more severe gambling disorder on both measures.

**Table 4:** Psychometric scores compared by the nature of the labels used for others

	Non-clinical label only (*n* = 59)	Clinical label (*n* = 241)	t-value	Cohen’s d
NODS	4.81 (2.69)	6.91 (2.54)	6.53***	0.80
PGSI	8.19 (5.67)	12.78 (6.89)	5.35***	0.73
GPSS	38.36 (8.10)	41.71 (6.86)	3.20**	0.45
GESS	27.54 (7.31)	32.85 (8.34)	4.14**	0.68

*N* = 270. GPSS = The Gambling Perceived Stigma Scale; GESS = The Gambling

Experiences Stigma Scale

Given that both severity and stigma were related to whether people adopted clinical labels, a logistic regression was conducted to examine whether stigma predicted the adoption of clinical labels after controlling for PGSI scores on the first step of the analysis. Two models were run: one for GPSS and another for GESS. In both models, the stigma variable was found to be non-significant once PGSI scores had been controlled.

## Discussion

The results provided a number of important insights into how people label and attribute the causes of their gambling problems. The first finding was that people are most likely to adopt self-labels that describe their condition as a form of addiction or ‘problem’; they are much less likely to use the common public health terminology which includes references to harm. A second finding was that people appear to modify the labels when describing themselves to others. Terms such as ‘problem’ or ‘compulsive’ are rarely used and only one in five endorsed the public health or ‘harm-related’ labels. Instead, by far the most strongly endorsed term was ‘addicted’ (i.e., that the person had an addiction). A third finding was that endorsement of labels (and this includes most clinical and harm labels) tended to be stronger when people had more severe problems with gambling as based on higher PGSI scores. People with more severe gambling problems also appeared to report greater perceived as well as public stigma. A fourth area of analysis related to the causal attributions which people make for their gambling problems and how these related to other variables. The findings showed that over 90% of people were willing to admit that their own actions were a cause of their problems (usually along with other factors) but, contrary to predictions, one form of stigma was found to be lower when people make more internal attributions (i.e., own actions > other external causes) for their gambling problems. Finally, it was found that people who adopted more clinical labels when describing themselves to others reported higher stigma scores; however, this effect dropped out once the severity of gambling problems had been controlled in the model. In other words, there was no compelling evidence that stigma was higher if people attributed their gambling problems to their own actions or used more clinical labels when describing themselves to others.

The fact that people are willing to attribute their problems to their own actions and to adopt labels suggests that fears about “over-pathologisation” may need to be contextualised. The type of language and approach to understanding gambling-related problems may differ depending upon whether the context is public (e.g., in marketing, help-services) as opposed to private (e.g., a person in treatment). As Penfold et al. (2024) note, terms such “addiction” may, in clinical and counselling contexts, be a useful way for people to understand and explain their condition to others. It attributes blame to a process or condition rather than any moral failure on the part of the individual. In this way, it provides a lay person’s way of making sense of their behaviour and potentially underscores the potential value of treatment. People appear much more likely to find this a useful label than ones relating to ‘harm’ which is a more indirect term which addresses the consequences of the gambling, but not the actual causes (the behaviour). Internal attributions or problem recognition is also a central feature of *Protection Motivation Theory* (Rogers, 1975) which states that problem recognition is a central feature of behavioural change and help-seeking. Problem recognition is also a fundamental element of motivational interviewing and involves helping individuals acknowledge a problem and the need for change, which usually assumes changes in attitudes and behaviour rather than an externalisation of the causes (Forman et al., 2025). For example, it may be that in some clinical or therapeutic contexts that people feel a sense of empowerment or agency when they admit to themselves that ‘they have a gambling problem’, ‘an addiction’ or ‘might be a problem gambler’, but this context needs to be distinguished from the broader public context where such labelling (especially by others) may lead to shame, withdrawal and defensiveness. Recognition also needs to be made of individual differences. Those who internalise negative public opinions and who develop a sense of self-shame through seeing themselves as ‘a problem’ may have poorer therapeutic outcomes (Horsh & Hodgins, 2015).

Several findings do, however, emphasise the potential importance of stigma. On the whole, scores on both stigma measures were higher when people had more severe gambling problems. These findings support the view reported in other papers (e.g., Hing et al., 2016; Quigley et al., 2020) that gambling problems make people fearful of being judged negatively by the public and that this fear may be increasingly internalised as the person’s problem develops. There may be greater feelings of shame, a desire for concealment, depression and anxiety and a greater sense of social distance from others. The complexity, as we found in this study, is that people may tend to adopt more clinical labels and self-pathologize themselves as their condition gets worse, and this was related to greater stigma. However, the findings suggest that this may be confounded by the greater severity of the condition. In other words, having a more severe gambling problem is probably a common antecedent of a greater sense of stigma and clinical labelling rather than stigma arising from the label. Nevertheless, there are some findings which do support the view that labels are important. People are much more likely to use a variety of clinical labels to describe themselves, but they are much less likely to use the same labels when describing themselves to others (e.g., problem, pathological or compulsive). The only term which runs against this trend is ‘addicted’ which seems to be a term that resonated with individuals in this sample.

### Implications of the Findings

The findings broadly support the view that labels and language is important when describing people with gambling problems. In public health contexts where there may be a desire to encourage people to seek help, avoidance of a number of the clinical terms appears to be sensible. Many people do not appear to endorse terms such as ‘problem’ or ‘pathological’ to others and are unlikely to respond favourably to them in public health promotions. However, the findings suggest that terms such as ‘people harmed by gambling’ (commonly now adopted by many governments) are not commonly used by people negatively affected by gambling. Instead, the term ‘gambling addiction’ would seem to be a more strongly preferred term. Use of this term does not appear to be strongly related to greater stigma once one controls for problem gambling severity and this supports the earlier findings of Quigley et al. (2020). For this reason, a term such as ‘person with a gambling addiction’ may be a preferable term that strikes the right balance between academic understanding and attempts to reduce stigmatising language.

The findings encourage more balanced and nuanced debates about the use of language in the field of gambling studies. For example, while we support the avoidance of some clinical or negative terms (e.g., ‘problem’ and ‘pathological’) in practical public policy, help-seeking and some applied contexts, we question whether the preoccupation with language in some more academic contexts is justified. Given that many people with gambling problems appear to use this language when self-labelling, it seems difficult to understand why they would be offended if such language were used in scientific papers or in clinical contexts (e.g., ‘addicted’ or ‘pathological or problem gambler’). Such language also accurately reflects the clinical nature of the disorder (i.e., addiction or pathology). To avoid it would almost appear to be a form of political correctness and censorship. Indeed, when the context is switched from academic classification to treatments, it seems odd for the language to be about gambling harm (the consequences of the behaviour) once people have sought help and the focus is on changes in behaviour. Finally, across both contexts there it seems almost unhelpful and out-of-step with the views of the population affected to avoid references to individual responsibility in the development of gambling problems. Many people do appear to admit that their own actions led to the problems, but that their behaviours needs to be viewed in the context of broader external factors (e.g., gambling accessibility, risky products). For this reason, use of the term ‘gambling addiction’ helps to understand the cause of the problem and that the pathway out of their situation also lies in their own hands, albeit with the supports of others. It also, as Delfabbro and King (2020) have noted, may prove strategically useful in legal contexts where sentencing mitigation may be more likely if the gambling client can be shown to have an illness, impairment of judgement, or an inability to control behaviour. Simply saying that they were ‘harmed’ is unlikely to achieve the same objective.

### Limitations

It is important to acknowledge a number of limitations of this study. First, it is reliant on a screened but self-selected sample of people and uses a self-report methodology that is vulnerable to biases so it is not clear whether the findings reflect people’s actual views. Second, the study involved an online panel and would need to be replicated using other populations. Third, some of the findings involved retrospective recall of gambling experiences which may have differed at the time they actually occurred. Fourth, we also acknowledge that the study is not conducted within a real-life public health context where people may be making real-life decisions about the need to seek help. A final point, identified by a reviewer, is that the new public health language has not been ‘socialised’ or promoted to the public for very long. As a result, it might therefore not be surprising to find that people do not endorse the ‘harm based’ language very strongly. Nevertheless, the results still show that clinical language still appears to resonate quite strongly and terms such as ‘pathological’ gambler are very academic and clinical terms which are not likely to have been used by laypeople. In other words, the preference for clinical terms is unlikely to be solely due to the greater longevity of these terms.

## Conclusions

This study showed that people are most likely to see gambling-related problems as being a form of addiction. Newly developed public health language relating to gambling harm does not appear to resonate as strongly with people who have experienced problems with gambling. We acknowledge that this may be due to the fact that some terminology is relatively new and less linguistically ‘accessible’ when people have to find a way to describe their actions to themselves and others. However, we have concerns that a sole focus on gambling harm and related terminology may not always be useful in many contexts where the interest might be on changing people’s behaviour (e.g., in harm minimisation strategies) or in tracking progress against clinical goals where control over behaviour may be a central predictor of reductions in harm. In such contexts, progress will likely be indicated by changes in the nature of the person’s gambling before any resultant reductions in harm which can sometimes take time to occur. Our research also shows that stigma, while related to the severity of gambling disorders, it does not appear to be strongly related to the language which is used by people to describe their condition. We believe that further research is needed to replicate this work using different populations and in different contexts (public and clinical samples) and to examine to what extent variations in labelling and language affect the appeal to services and help-seeking.

## Declarations

Tyler MacGinlay. No declarations

Paul Delfabbro. Over the last 3 years, I have undertaken consultancy and research work for regulatory organizations (NZ Gambling Commission); government departments (South Australian Department for Human Services) and conducted views of product risk for industry groups including Premier Lotteries Ireland; analysed the relationship between product engagement and risk indicators in project in a project funded and supported by from Kindred Pty Ltd; and, conducted reviews of product risk and vulnerable populations for Allywn in the UK.

Daniel King. I have undertaken consultancy and research work related to gaming disorder, gaming-gambling convergence, and adolescent digital technology use for the World Health Organisation and various government departments in Australia and I have received honoraria for conference and seminar presentations in the past 3 years. I have no gaming- or gambling-related industry connections or related commercial relationships to declare.

Date Statement. The full data-set is available from the authors upon request.

## References

[CR1] Barney, L. J., Griffiths, K. M., Jorm, A. F., & Christensen, H. (2006). Stigma about depression and its impact on help-seeking intentions. *Australian and New Zealand Journal of Psychiatry*, *40*, 51–54. 16403038 10.1080/j.1440-1614.2006.01741.x

[CR2] Brown, K. L., & Russell, A. M. T. (2020). What can be done to reduce the public stigma of gambling disorder?? Lessons from other stigmatised conditions. *Journal of Gambling Studies,**36*(1), 23–38. 31520273 10.1007/s10899-019-09890-9

[CR3] Browne, M., & Rockloff, M. J. (2020). Measuring behavioural dependence in gambling: A case for removing harmful consequences from the assessment of problem gambling pathology. *Journal of Gambling Studies*, *36*(4), 1027–1044. 31776754 10.1007/s10899-019-09916-2

[CR4] Corrigan, P. W., & Watson, A. (2002b). The paradox of self-stigma and mental illness. *Clinical *

[CR5] Delfabbro, P., & King, D. L. (2020). Don’t say the ‘P’ word: Problem gambling is more than harm. *International Journal of Mental Health and Addiction,**18*(3), 835–843. 10.1007/s11469-020-00274-4

[CR6] Donaldson, P., Langham, E., Best, T., & Browne, M. (2015). Validation of the gambling perceived stigma scale (GPSS) and the gambling experienced stigma scale (GESS). *Journal of Gambling Issues*, 163–200. 10.4309/jgi.2015.31.8

[CR7] Evans, L., & Delfabbro, P. (2005). Motivators for change and barriers to help-seeking in Australian problem gamblers. *Journal of Gambling Studies*, *21*(2), 133–155. 10.1007/s10899-005-3029-4 15870984 10.1007/s10899-005-3029-4

[CR8] Ferris, J., & Wynne, H. (2001). *The Canadian problem gambling index*. Canadian Centre on Substance Abuse.

[CR9] Forman, D. P., Boughter, J. R., McAfee, N. W., Ginley, M. K., Whelan, J. P., & Pfund, R. A. (2025). Motivational-interviewing-informed interventions for problem gambling and gambling disorder: A systematic review and meta-analysis. *Psychology of Addictive Behaviors,**39*(4), 365–374. 10.1037/adb0001069 40372876 10.1037/adb0001069

[CR10] Galanis, C. R., Weber, N., Hing, N., Delfabbro, P. H., Billieux, J., & King, D. L. (2025). Stigma in substance-based and behavioural addictions: A systematic review. *Journal of Behavioral Addictions*, *14*, 79–99. 10.1556/2006.2024.00072 39819679 10.1556/2006.2024.00072 PMC11974440

[CR11] Gernstein, D., et al. (1999). *Gambling impact and behavior study: Report to the National gambling impact study commission*. Massachusetts.

[CR12] Hing, N., & Russell, A. M. T. (2017). How anticipated and experienced stigma can contribute to self-stigma: The case of problem gambling. Frontiers in psychology, 8 (FEB), art. 235.10.3389/fpsyg.2017.00235 10.3389/fpsyg.2017.00235 PMC5318456 28270787

[CR13] Hing, N., Nuske, E., Gainsbury, S. M., & Russell, A. M. T. (2016). Perceived stigma and self-stigma of problem gambling: Perspectives of people with gambling problems. *International Gambling Studies*, *16*(1), 31–48. 10.1080/14459795.2015.1092566

[CR14] Horch, J. D., & Hodgins, D. C. (2015). Self-stigma coping and treatment-seeking in problem gambling. *International Gambling Studies*, *15*(3), 470–488.

[CR15] Langham, E., Thorne, H., Browne, M., Donaldson, P., Rose, J., & Rockloff, M. (2016). Understanding gambling related harm: A proposed definition, conceptual framework, and taxonomy of harms. *BMC Public Health,**16*, 80. 26818137 10.1186/s12889-016-2747-0 PMC4728872

[CR16] Leslie, R. D., & McGrath, D. S. (2024). Stigma-related predictors of help-seeking for problem gambling. *Addiction Research and Theory 32,* 38–45. 10.1080/16066359.2023.2211347

[CR17] Lloyd, J., Penfold, K. L., Chadwick, D. D., Nicklin, L. L., Hinton, D. P., & Dinos, S. (2025). Stigmatisation of people experiencing gambling-related harms: A vignette study of the predictors of desire for social distance. *Frontiers in Psychology,**16*, Article 1613798. 10.3389/fpsyg.2025.1613798 40606865 10.3389/fpsyg.2025.1613798 PMC12213880

[CR18] Palmer, B. A., Richardson, E. J., Heesacker, M., DePue, M. K. (2018). Public stigma and the label of gambling disorder: Does it make a difference? *Journal of Gambling Studies, 34*(4), 1281-1291. 10.1007/s10899-017-9735-x 10.1007/s10899-017-9735-x 29243011

[CR19] Penfold, K., Nicklin, L. L., Chadwick, D., & Lloyd, J. (2024). Gambling harms, stigmatisation and discrimination: A qualitative naturalistic forum analysis. *PLoS One,**19*(12 December), Article artnoe0315377. 10.1371/journal.pone.0315377 10.1371/journal.pone.0315377 PMC11630613 39656711

[CR20] Quigley, L., Prentice, J., Warren, J. T., Quilty, L. C., Dobson, K. S., & Hodgins, D. C. (2020). What’s in a name?? Evaluating the public stigma of gambling disorder. *Journal of Gambling Studies,**36*(4), 1205–1228. 10.1007/s10899-019-09924-2 31848837 10.1007/s10899-019-09924-2

[CR21] Rogers, R. W. (1975). A Protection Motivation Theory of Fear Appeals and Attitude Change1. *The Journal of Psychology*, *91*(1), 93–114. 10.1080/00223980.1975.9915803. 10.1080/00223980.1975.9915803 28136248

[CR22] Rundle, S. M., Goldstein, A. L., Wardell, J. D., Cunningham, J. A., Rehm, J., & Hendershot, C. S. (2025). Examining the relationship between public stigma, models of addiction, and addictive disorders. *Addiction Research and Theory*, *33*(2), 143–149. 10.1080/16066359.2024.2365156 40453885 10.1080/16066359.2024.2365156 PMC12124819

[CR23] Victorian Responsible Gambling Foundation. (2024). *). Reducing stigma. A guide for talking about gambling harm*. Melbourne.

[CR24] Wöhr, A., & Wuketich, M. (2021). Perception of gamblers: A systematic review. *Journal of Gambling Studies,**37*(3), 795–816. 10.1007/s10899-020-09997-4 33660191 10.1007/s10899-020-09997-4 PMC8364520

